# Circulating osteopontin: a dual marker of bone destruction and angiogenesis in patients with multiple myeloma

**DOI:** 10.1186/1756-8722-4-22

**Published:** 2011-05-08

**Authors:** Aikaterini Sfiridaki, Spiros Miyakis, Constantina Pappa, George Tsirakis, Athanasios Alegakis, Vasileios Kotsis, Efstathios Stathopoulos, Michael Alexandrakis

**Affiliations:** 1 Blood Bank Center, Venizelion General Hospital Heraklion, Crete, Greece; 2 Third Department of Medicine, Aristotle University of Thessaloniki, Papageorgiou General Hospital, Thessaloniki, Greece; 3 Department of Hematology, University Hospital of Heraklion, Crete, Greece; 4 Toxicology Laboratory, Medical School of Crete, Greece; 5 Department of Pathology, Medical School of Crete, Greece

## Abstract

The matrix protein osteopontin has been shown to be a marker of osteoclastic activity in multiple myeloma patients, as well as a regulator of angiogenesis. We measured serum levels of osteopontin in 50 untreated multiple myeloma patients (in 25, also after treatment) and examined the relation to markers of osteolytic and angiogenic activity. The median (range) of serum osteopontin was 85 (5-232) in the patient group vs. 36 (2-190) ng/ml in the control group. Serum osteopontin levels were significantly higher in patients with advanced stage or grade of myeloma disease. All patients with serum osteopontin levels >100 ng/ml had advanced stage (II or III) or high grade bone disease, whereas stage I or low grade patients had serum osteopontin levels <100ng/ml. Serum osteopontin levels significantly decreased after treatment. There was a positive correlation of osteopontin with the bone turnover marker N-terminal propeptide of procollagen type I (NTx) and the angiogenic markers vascular endothelial growth factor (VEGF) and bone marrow microvessel density (r: 0.35, 0.47 and 0.30 respectively, p < 0.05). These results support osteopontin as a dual marker of bone destruction and angiogenic activity in myeloma patients. Osteopontin represents a useful biomarker for monitoring myeloma disease activity.

## To the Editor

Osteopontin (Opn) is a non-collagenous matrix protein responsible for the migration and attachment of osteoclasts to mineral matrix of bone surfaces [1]. I*n vitro *studies suggest that Opn and vascular endothelial growth factor (VEGF) cooperatively enhance angiogenesis in MM [2,3]. We compared the serum levels of Opn with those of established markers of angiogenic activity and bone destruction in 50 (23 males, median age 68 y.o.) untreated MM patients and 25 healthy age & sex-matched blood donors (median age 65 y.o.). In 25 patients, measurements were repeated after reaching plateau phase with chemotherapy. Ethics approval and informed consent from all subjects were obtained.

Osteopontin and VEGF were measured with a solid-phase sandwich enzyme-linked immunosorbent assay (Quantikine^®^, R&D Systems Inc. Minneapolis MN, USA). Bone marrow cellularity and percentage of infiltration by myeloma cells was estimated in all patients. Microvessel density (MVD) was assessed on bone marrow sections after staining endothelial cells with the anti-CD34 antibody (Immunotech, Marseille, France) [4]. The N-terminal propeptide of procollagen type I (Ntx) in urine was measured by a competitive inhibition ELISA (Ostex International, Seattle, WA, USA) [5]. Classification and regression tree (CART; Salford Systems, San Diego, CA, USA) analysis was used to identify the Opn levels best associating with disease parameters.

**Figure 1:**
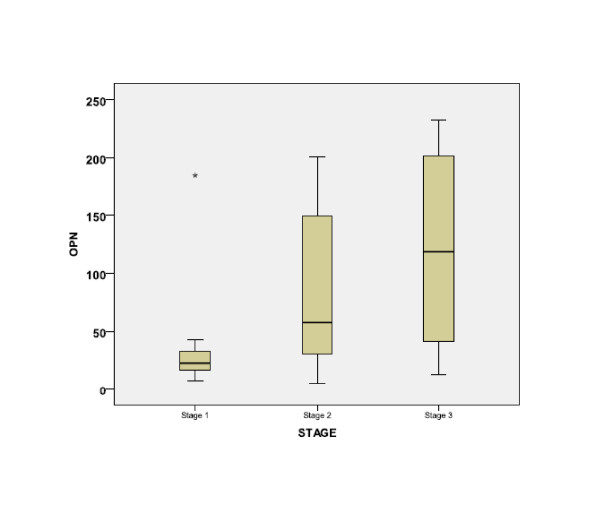
**Serum osteopontin levels (ng/ml) according to Durie-Salmon stage in multiple myeloma patients**. Columns: 25-75% of values; bold line: median; whiskers: 95% confidence intervals. (* = p < 0.01 by Kruskal Wallis test).

**Figure 2:**
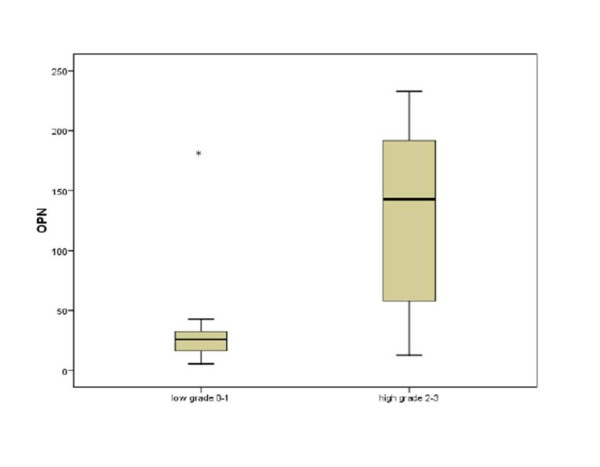
**Serum osteopontin levels (ng/ml) according to grade of bone disease (low grade = grades 0 and 1; total 23 patients, versus high grade = grades 2 and 3; total 27 patients)**. Columns: 25-75% of values; bold line: median; whiskers: 95% confidence intervals. (* = p < 0.01 by Kruskal Wallis test).

Opn among MM patients [median (range) 85 (5-232) ng/ml] was higher than controls [36 (2-190) ng/ml] but the difference did not reach statistical significance. However, Opn was significantly higher in 35 patients with Stage II or III compared with 15 Stage I patients (p < 0.001) (Figure 1). Similarly, there was a marked difference in Opn according to disease Grade (Figure 2), with increasing levels from Grades 0 to 3 (p = 0.006).

CART analysis determined Opn >100 ng/ml as predictor of advanced MM disease. All 17 patients with Opn >100 ng/ml had advanced stage (II or III), whereas all 15 stage I patients had Opn <100 ng/ml (Odds Ratio 1.5, 95% Confidence Intervals 1.2-2.0; p = 0.036 Fisher's Exact test). Similarly, high grade (2 or 3) MM was associated with Opn >100 ng/ml (OR 1.9, 95% CI 1.2-2.9, p = 0.001) (Table 1).

**Table 1:** Association of serum osteopontin levels with: A) stage, and B) grade of Multiple Myeloma

Multiple myeloma status	Serum osteopontin level (ng/ml)		OR (95% CI)
	<100	≥ 100	
A. **Stage**			
Stage 1	15	0	1.5 (1.2-2.0)
Stage 2-3	18	17	

B. **Grade**			
Low (0-1)	23	0	1.9 (1.2-2.9)
High (2-3)	15	12	

Serum osteopontin cut-off has been determined using classification and regression tree analysis.

OR = Odds Ratio; CI = Confidence Interval.

Opn correlated significantly with VEGF, MVD, Ntx and disease infiltration (Spearman's rho 0.47, 0.30, 0.35 and 0.45 respectively; p < 0.05). In 25 patients, Opn decreased from a median 78 ng/ml to 45 ng/ml post-chemotherapy (p < 0.05). MVD, Ntx, VEGF and infiltration by myeloma cells were also significantly reduced post-treatment (p < 0.001).

Our results add to the weight of data supporting a relationship between circulating Opn and bone marrow angiogenesis in myelomatous disease. We have defined a cut-off value (100 ng/ml) strongly associated with advanced MM disease and grade of bone destruction. This may prove useful in patient stratification. The applicability of our findings in different patient populations (i.e. genetic background, treatments used, comorbidities, etc) awaits validation.

## List of Abbreviations

MVD: microvessel density; VEGF: Vascular-Endothelial Growth Factor; VEGFR2: Vascular-Endothelial Growth Factor Receptor 2; Ntx: N-terminal propeptide of procollagen type I; ELISA: enzyme-linked immunosorbent assay.

## Conflict of interest

The authors declare that they have no competing interests.

## Authors' contributions

AS performed the experimental procedures; SM collected patient data and samples, assisted with statistical analysis and data interpretation, and wrote the manuscript; CP collected patient data and samples; GT collected patient data and samples; AA performed the statistical analysis; VK assisted with data interpretation and writing of the manuscript; ES performed the pathology measurements used in the study; and MA designed and coordinated the study, and interpreted data.

All authors have read and approved the final manuscript.
